# Supplementary material for: Where and what? Frugivory is associated with more efficient foraging in three semi-free ranging primate species
Source: R Soc Open Sci. 2019 May 1;6(5):181722. doi: 10.1098/rsos.181722 (PMC6549983; doi:10.1098/rsos.181722)
Supplement: Supplementary material (ESM) [file rsos181722supp1.pdf]

Royal Society Open Science - Supplementary materials for

"Where and What? Frugivory is associated with more efficient foraging in three semi-free ranging primate species"

Cinzia Trapanese, Benjamin Robira, Giordana Tonachella, Silvia di Gristina, Hélène Meunier*, Shelly Masi*

*equal contribution

This supplementary file includes:

1) Materials & Methods (Hierarchical relationships, Food preference test, Habituation to the experimental protocol, Test Phase)

2) Statistical analysis (Food preference test, Models' goal, Model's sample, Model selection, Models' stability, Models implementation)

3) Results (Food preference test, Index of Lateralisation)

Supplementary Tables S1-S10

Supplementary Figures S1-S5

Supplementary Bibliography

1) Materials & Methods

Experimental rooms that allow temporarily separation of a subject from the rest of the group are connected to each park by sliding doors (outdoor area: *M. Tonkeana*=3788m^2, *M. fascicularis*=1364m^2 and *S. apella*=2332m^2; Figure S1a, b, c). Individuals had free access to both outdoor and heated indoor areas, the latter connected to the former.

Hierarchical relationships

For each group, we assessed the hierarchical relationships among the individuals using a competition test allowing to assess the individual social rank [S1]. We recorded all occurrences

[44] of the dyadic agonistic interactions (aggressive displays by an individual and the recipient reaction to it) for drinking from a single source of fruit syrup (10% diluted in water) fixed at the mesh of the enclosure of each study group. Specifically, aggressive behaviour was defined as any silent or vocal facial threat, lunge, chasing, pushing, hitting, grabbing, biting, flighting, cowering, screaming and counteraggression. We also recorded submissive behaviours as avoidance [S2]. To determine the robustness and the statistical significance of the linearity in the observed relationships, we carried out hierarchical rank order analysis using the software Matman, edited by Noldus, and calculated the improved index of linearity [S3, S4].

Food preference test

We used eight different type of appealing domestic fruit with fairly comparable nutritional values: with *M. tonkeana* dry raisin, mango, pineapple, banana, orange, pear, melon and avocado have been used, while with *M. fascicularis* and *S. apella* kiwi, orange, pineapple, mango, banana, watermelon, pear and apple (Table S4). Fruits were cut in pieces of 1cmx1cm with macaques and 0.70cmx0.70cm with *S. apella*, scaled according to their body size. At each of the 28 dicotomical choices [S5], subjects had to choose one of the two fruit types by unequivocally touching one hand of the experimenter, each hand holding a piece of fruits. A daily session of 28 trial choices was carried out with *M. tonkeana*, while to increase *M. fascicularis* and *S. apella* motivation for them the session was split in 14 trials in the morning and the other 14 trials in the afternoon. We carried out a total of 10 differently randomised sessions for each subject. The order of fruit preference was calculated using Matman software version 1.1.

Habituation to the experimental protocol

During the Test Phase, primates had to search for fruit inside 36 wooden boxes. Each box was associated with a single fruit type throughout the study. In an attempt to mimic the tree bark

texture design, a fruit specific *visual cue* was fixed under each box (a 40×23cm wooden engraved panel, Figure S3) to allow unique identification of each of the fruit species used. Prior to the Test Phase we carried out a Habituation Phase consisting of two main steps. The first step, the *habituation in the indoor area,* lasted five weeks and took place in the experimental rooms that are connected to the outdoor area by sliding doors. The individuals that voluntary entered into the experimental room were isolated and tested. The aim of this first step was to teach to primates which box was available, thus openable, and when they could open it.

At first, only one box without *visual cue* with *M. tonkeana* and *S. apella* was fixed in the experimental room at 1m high already filled with fruit (as it would be for the Test Phase). Since *M. fascicularis* tended to be neophobic, for them two boxes were fixed almost at the ground level to facilitate the exploration. We used a very attractive fruit (dry raisin) that was not used during the Test Phase. First, each focal animal had to understand that the trial started only when the experimenter showed them a personal starting cue (i.e. an object, Figure S4). To pass this stage of the Habituation the individuals had to look at the *personal starting cue* showed by the experimenter and successively open the box with the hands for at least four consecutive times. After that, all five boxes (one for each different task, with CvQ having two boxes with two different fruit types) with the correspondent visual cues were placed in the room (Figure S5). Primates had to understand that at each trial, only one box per trial was filled with the correspondent fruit and the same fruit was available for five consecutive days. The order of fruit availability during the Habituation Phase followed always the same temporal pattern: Spatial Foraging Task (discussed elsewhere, Trapanese et al. *in review*), Clumped vs Scattered Task 1 (CvS1), Clumped vs Scattered Task 2 (CvS2), Clumped vs Quality Task (CvQ). Also the temporal pace was always the same within a species: each fruit lasted five days (but

for *M. tonkeana* three days because of improvement of the experimental protocol after testing them) the same temporal pace of each fruit availability, thus of each task/season during the Test Phase. At each trial (N=60 trials per individual, 15 trials per fruit) the corresponding *food cues* (fruit's skins or peels) of the available fruit in the box was scattered on the ground. The food cues allowed subjects to learn which box contained fruit at each trial, i.e. which box could be opened. In all trials but the first, the boxes were baited out of the sight of the subjects. To let them understand that the box could be opened only when the experimenter showed them the *personal starting cue,* this latter was presented with a delay of time (from 5 sec to 2.45 min) that increased progressively of five or ten seconds each week (i.e. each new fruit presented) starting from the second week. The passing criterion of this part of the habituation was that after the signal of the *personal starting cue* the subject had to visit as first the baited box and have to open it in at least three consecutive trials for each of the five fruits. Among the 22 subjects who started the indoor habituation, 17 reached these criteria and thus could participate to the second part of the habituation, *the outdoor habituation* (see Table S3 for number of subjects for each species). In this second step, two experimenters were in the outdoor area together with the study group, one following the focal subject and the other one attracting the dominant(s) away like in the Test Phase. Starting from the second month, all 36 boxes from all tasks/seasons were in the outdoor area together with the corresponding visual cues (they were not removed over the weekends). Each week, the corresponding food cues of the task/season were scattered on the ground. Only during the first complete series of tasks/seasons of the Habituation Phase, subjects were attracted by the experimenter with food towards the area of the baited boxes, if necessarily. In this case, once the focal individual passed nearby a baited box, the experimenter opened that box using the remote system that makes a specific opening-sound that efficiently attracted the

attention of the focal individual. The criterion for passing this step was that each subject had to visit without any incitement or cue from the experimenter all boxes of each task/season in at least 10 trials during the first two seasonal completed series/tasks (roughly two months). Fourteen individuals out of 17 (see Table S3 for number of subjects for each species) passed this second part of the habituation and thus could participate to the Test Phase.

Test Phase

Fruits were provided in the boxes always in the same quantity of pieces of the same size as in the food preference test. To minimise the influence of social competition on the foraging choice of the more subordinate individuals (N=3 in *M. tonkeana*, N=2 in *M. fascicularis* and N=4 in *S. apella*), when possible one of the two experimenters attracted the dominant individuals with food in a side corner of the outdoor area that was the most distant from any boxes. When possible, we tested opportunistically the individual passing nearby the starting point or we called him/her from the starting point and waited until the individual was in the good position to start (equal distance from the two distributions, any point on the red line in Figure 1, 2 and 3). Each trial was considered finished if the focal individual stopped somewhere for more than ten minutes regardless if the individual already visited all boxes or not.

When we filled and pretended to fill the boxes, we first covered the top of it with a white cloth, then we opened it, we put the fruit inside and we closed the box with the remote control or imitate with fingers the sound of it in case of the fake filling of the boxes. We never tested a subject that saw us filling the last boxes (the last that we filled following always a different random order), neither a subject that was following the previous subject tested. It has never happened that a subject followed us or another subject for the entire route or trial.

2) Statistical analysis

Food preference test

The software Matman calculated the rank order of the eight fruits and the correspondent linear hierarchy index for dominance matrix. To choose the most and the least preferred fruit from all tested individuals we chose the 1st and the 5th preferred fruit following the order of preference of *M. tonkeana*. Since in this case one individual Nereis did not have the same group preference for the most and least preferred fruit thus we needed to exclude her from the analysis of the CvS1 and CvS2 (see paragraph "Models' stability" in ESM), the following year we improved the protocol for *M. fascicularis* and *S. apella*. An additional criterion was used to increase the difference in preference between the two fruit types for the Clumped vs Quality Task ensuring that all tested individuals had the same direction of preference: the most preferred fruit was the one chosen by each subject at least seven times in the ten sessions (of 28 choices), consequently the least preferred was the fruit chosen for maximum three times per individual.

Models' goal

In the "Successive five visited boxes" models we choose to consider the successive five visited boxes after the first visited box, and we included the visits to both baited and non-baited boxes made by any focal individual at each trial. We aim here to discuss the reasons that lead us to this choice of analysis. First, we considered the visited five boxes after the first box and not all successive visited boxes since such an approach, although very conservative and close to observational methods in the wild would have, however, triggered many issues such as:

a) Having differential sample across individuals, with individuals making erroneous choices (non-baited boxes), hence, those indicating memory lapse and/or failures, having a higher rate of observation, hence a higher weight within the model.

b) Several strategies might exist among individuals across a long series of boxes to open: e.g. back and forth movements, visit only one circular distribution (clumped or scattered) then

the other, visiting one circular distribution and if the error rate is too high then switching to other circular distribution. Hence, if constructing only one model, this one would have been too complex (and, potentially, impossible, as strategies might be mutually exclusive and/or impossible to describe with the same variables).

Secondly, we chose to take into account both visited baited and non-baited boxes. We could have considered only the visited boxes belonging to the current task/season (the baited ones) but such an approach would have been biased, as we wanted to see if their choice was robust, hence if they remained in the clumped circle. If excluding the non-baited boxes from the analysis, we would have excluded the information associated to them, and potentially missing the switches to the opposite distribution. In addition, considering only the visited baited box would have not truly depicted the effect of other influencing variables, such as the social variable (e.g. number of competitors), for the feeding choice. Last but not least, if we had considered only the baited boxes, the probability for a given individual to choose a successive box in the clumped distribution would have been much higher than to choose it in the scattered distribution. Thus, since we considered both baited and non-baited visited boxes, the probability to find another box (either baited or not baited) when being in either one of two distributions was identical (being the clumped and scattered distributions overlapping in space across tasks/seasons, Figure 1a, b, c).

Therefore, to reconcile all these views, we started from the idea of the pure optimal strategy of an omniscient individual: the individual will forage on the clumped distribution, and directly target the baited boxes. Therefore, we chose to consider only the six first boxes visited and modelled a) the probability of choosing the clumped distribution (using model “First visited box” and model “Successive five visited boxes”) and assess its robustness, b) the error rate within this choice (model “Goal-directed strategy”) and c) the propensity to leave the foraging

distribution. Surely, this approach also has bias but that we considered minor as compared to the other ones as bias described in (a), if considering all successive visited boxes) is reduced to the minimum: it is still present (although we discarded many possibilities, hence we still can describe the "first part" of the strategy, although not fully) and it has the advantage of having equal sample for all individuals. We tried to account for this with the analysis of the following behaviours (the box/boxes chosen after the 6th one), separately.

Always starting form an ideal individual (omniscient one) the last step was to investigate if primates had the spatial knowledge but also the temporal one: i.e. if once chosen the clumped distribution, and all baited boxes of that distribution, do they continue to visit non-baited boxes of that distribution? Do they stop to forage? Or in order to forage efficiently do they show temporal knowledge going to visit also the other available baited boxes, i.e. those of the scattered distribution? We thus ran the last model "Change of the distribution" to assess whether the probability to leave the current patch was linked to the nature of the previous visited box (baited or not) and/or its distribution (clumped or scattered). Finally, we calculated the percentage per species of the visits to at least three boxes of the scattered distribution, only in trials in which they visited all boxes of the scattered distribution. We decided to consider at least three boxes since they correspond to half of the circle, this allowed us to take into account that in provisioned primates some individuals may have had lower motivation for the second part of the Test.

Model's sample

To assess the effect of the distribution on the choice, and then of the quality, we subsequently ran the models on either task CvS1 and CvS2 (model "First visited box CvS" and "Successive five visited boxes CvS"), or, on CvS1 and CvQ (model "First visited box CvQ" and "Successive five visited boxes CvQ"). We did so because CvS1 and CvQ had the same location of the

clumped and the scattered distributions regarding the configuration in relation to the side of the outdoor area. For those, as the result depended on the choice, we only included individuals for which the intensity, or the lateralisation *per se* namely the preference for a side of the outdoor area because of presence of other individuals around the entrance to the indoor area (rather than a preference for a fruit distribution) changed between CvS1 and CvS2. Therefore, we excluded by these analyses the following individuals: Laeticiette (*M. fascicularis*) and Willow (*S. apella*).

With regards to model “Goal-directed strategy”, the output variable was internal to the choice and no longer depended on it: it only assessed how much primates tried to open boxes from the non-baited distributions (of other tasks/seasons), depending on the state of the analysed variables. Therefore, we included in the analysis also the two individuals previously excluded despite their unique preference without change in intensity for a given site of the outdoor area. In model “First box visited CvQ” and “Successive five visited boxes CvQ”, we also excluded Nereis (*M. tonkeana*), as her preferred food item was in the boxes of the clumped distribution, and the least preferred one was in the scattered distribution since her personal food preference was opposite to the group one (that we used to construct the experimental settings). She therefore did not match the working scenario of the experiment, and we hence removed her from these two analyses. This issue did not happen with any other individual. In models “Change distribution” and “Goal-directed strategy” , we included Nereis despite testing for the variable task as we wanted to test for the settings of the experiment (with the clumped and scattered distribution being localized at varying side of the enclosure accordingly to the task) and because we hypothesized that the quality of the food would not affect the error rate or the probability to change distribution. However, in order to exclude any doubt,

we also ran these two models without Nereis, and we did not found any difference in the significative results.

Model selection

Each time, we started with the model of highest complexity based on our hypothesis and knowledge. We then never modified the structure of the model itself (i.e. we kept all independent variables from start to the end), but only removed, terms of highest complexity (i.e. interactions) that were not significant (therefore, not meaningful), and of lowest AIC to limit risks of over parametrisation. In the main text, we explained the reasons of the structure of the model we started with, but then presented and discussed the results of the simplified models we ended up with.

Models' stability

To assess the behaviour of our models, namely its sensitivity to potential outliers/leverage points and/or individuals, we inspected the models' robustness and stability using several statistical indicators directly on the models including random effects (i.e. GLMMs, e.g. over/underdispersion; stability with regards to individuals' response) or on models excluding random effects (i.e. GLMs; e.g. DFbetas, leverage, cook's distance, Tables S7, S9, S10). Although conducting analyses on models without random effect might partially be representative of the true model's behaviour, it allowed us to broader the diversity of statistical indicators possible to use, and could allow us to diagnose potential issues, while we acknowledge that we might miss some if only referring to this approach, explaining why we use it as a complement of GLMM investigations. Visual inspection did not suggest outliers (not shown). Overall, leverage indicated only a few potential leverage points (values obtained for the presented models in the end: leverage max: 0.139, ratio leverage/ threshold max: 4.293, reached for model "Successive five visited boxes CvS", with the theoretical threshold being

2*(k+1)/n, with k being the number of predictors, n the number of observations), though Cook's distances did not point out such issue (Cook's distance max: 0.087, reached for model "First box CvQ"). Also, dfbetas on those equivalent GLMs indicated that those points did not affect estimations overall (Table S10). Troubles were at first at its paroxysm with model "Successive five visited boxes CvQ", for which worries were due to two choices of the individual "Tempete" (*M. fascicularis*) and because of the individual "Nereis" (*M. tonkeana*). We noticed that those two individuals corresponded to individuals who did not do the food preference test (i.e. Tempete), or, more critically, to an individual who had actually opposite preference with respect to that of the group in CvQ task (i.e. the least/most preferred food was actually the most/least preferred food respectively with Nereis). With regards to *M. fascicularis*, to which Tempete belongs, we adopted a null approach, assuming that her choices were in correspondence with those at the group scale, as we did not see considerable individual variations within the *M. fascicularis* tested for food preference (N=6). However, we chose not to consider Nereis, for which food preference did not suit the purpose of the CvQ task, when proceeding to model "Successive five visited boxes CvQ") analyses (but we kept her in model "Goal-directed strategy", as explained in the main corpus). We only present those later results, and therefore, when we evoke stability/robustness of the model, we refer to this latest sample. Note that deleting Nereis did not greatly improved model's robustness (model "Successive five visited boxes CvQ") though, as aforementioned statistical indicators still indicated few influential singular choices, which spread across several individuals. In the remaining models, only model "Goal-directed strategy" indicated some minor influential points. Again, we saw no reason of removing them. It did not improve statistical indicators which still pointed out leverage points, nor affected estimates importantly and changed significance of the tests.

We also investigated models' robustness based on models' over/underdispersion and models' stability (i.e. estimate variation while excluding for one level of the random factor at a time), this later allowing us to investigate the influence of each individual on the model outputs. Dispersion checks indicated no major worries (dispersion $factor_{min}$=0.924, dispersion $factor_{max}$=1.006). Stability did not point out major global issue (see Table S10).

Models implementation

To limit risks of over parameterisation, we removed terms of highest complexity (i.e. interactions) that were not significant and had the lowest AIC but conserving the variables independently. After deep investigation, reduced sample size did not seem to influence the models but results of the models' stability investigations underline that interpretations of the intensity of the observed behaviours must sometimes remain cautious (details in ESM "Models' stability").

Finally, when categorical predictors included more than two levels and had significant influence, we repeated the model analysis changing the reference level with categorical not of interest variables, manually dummy coded and centred, in order to obtain a post-hoc comparison to neutral choice (i.e. intercept significance) or between-level comparisons illustrated in figures.

Only in one model (model "First box visited CvQ", did we observe that excluding for low observation associated-values (i.e. here corresponding to more than three competitors) could lead to potential effect of rank and species, though both had been identified as false positive based on Benjamini and Liu's procedure. Therefore, we do not further discuss this aspect.

3) Results

Food preference test

We recorded a total of 2240 choices for *M. tonkeana* (28 binary combinations x 10 sessions per subject x 8 individuals), 1680 choices for *M. fascicularis* (28 binary combinations x 10 sessions per subject x 6 individuals) and a total of 2240 choices for *S. apella* (28 binary combinations x 10 sessions per subject x 8 individuals). We obtain the following group ranking of species' food preferences: *M. tonkeana*, raisin, mango, pineapple, pear, banana, orange, avocado, melon (Landau's index of linearity, h=0.9; Kendall's coefficient of linearity, K=0.9; p=0.001); *M. fascicularis*, banana, orange, pear, mango, pineapple, kiwi, apple, watermelon (h=0.96, K=0.96, p<0.001); *S. apella*, watermelon, mango, pear, pineapple, apple, kiwi, banana, orange (h=0.89, K=0.89, p<0.01; see Table S4). We calculated the order of preference considering all subjects that participated to the food preference test (N=22, Table S3 for details), before starting the Habituation Phase, in order to choose the fruit to use in each task/season. All subjects did not pass the criteria of the Habituation Phase and thus not all of them participated to the Test Phase (see the paragraph "Habituation to the experimental protocol" above and Table S3). Even if raisin was the most favourite fruit for *M. tonkeana* we decided to not use it because a) it was too different from the other fruit types in terms of dryness and nutritional values, and b) it would not be impossible to use non-appealing parts (e.g. skin or peel) for food cues to scatter on the ground during the Test Phase. In order to choose the most and the least preferred fruit for the CvQ task/season for each tested individual in *M. fascicularis* and in *S. apella*, we calculated for which pair of fruit types was true the minimum choice ratio 7:3. Orange and watermelon were chosen for *M. fascicularis* and pear and orange for *S. apella* (see Table S5 and S6 for fruit chosen for each species).

Index of Lateralisation

Among the five individuals unilateralised, two did not vary the intensity of the preference for one side when comparing CvS1 and CvS2 task (proportion test; Laeticiette χ^2= 2.182, df=1,

p=0.139, Willow χ^2= 1.144e-30, df=1, p=1). On the contrary, Nicolette and Yannick, also unilateralised towards one side of the enclosure (see Table 2a, b) had a clear change in preference's intensity between the contexts, hence we assumed a clear effect of food distribution and kept them for further analyses (proportion test; Nicolette χ^2= 15.916, df=1, p=<0.001, Yannick χ^2= 5.865, df=1, p=0.015).

Supplementary Tables

Table S1: Socio-ecology features of the study species.

Species	Diet (%)	System	Social tolerance
Macaca tonkeana	Fruit 85.8 %, young leaves 4.2 %, flowers 0.8 %, shoots 3.1 %, invertebrates 6.1 % [35]	Terrestrial [S6]	Resident-Nepotistic-tolerant [36]
Macaca fascicularis	Fruit 66.7 %, leaves 17.2 %, flowers 8.9 %, seeds, barks, fungi, insects 4.1 %, small invertebrates (especially crustaceans) 3.1 % [S7, 37]	Semi-arboreal [37]	Resident-Nepotistic; despotic [36]
Sapajus apella	Fruit 53.9 %, seeds 16 %, insects and small invertebrates 12.7%, flowers 11.1 %, leaves 6.3 % [38]	Arboreal [S8]	Resident-Nepotistic; tolerant [39]

Table S2: Name, species, sex, age and rank of the subjects who participated to the Test Phase.

ID subject	Species	Sex	Age (years)	Status
**Olli**	*Macaca tonkeana*	m	6	low-ranking
**Yannick**	*Macaca tonkeana*	m	7	high-ranking
**Walt**	*Macaca tonkeana*	m	9	low-ranking
**Wallace**	*Macaca tonkeana*	m	9	high-ranking
**Nereis**	*Macaca tonkeana*	f	17	high-ranking
**Tempete**	*Macaca fascicularis*	f	6	high-ranking
**Laeticiette**	*Macaca fascicularis*	f	9	low-ranking
**Nicolette**	*Macaca fascicularis*	f	14	low-ranking
**Koli**	*Sapajus apella*	f	5	low-ranking
**Franklin**	*Sapajus apella*	m	7	low-ranking
**Litchi**	*Sapajus apella*	f	8	low-ranking
**Willow**	*Sapajus apella*	f	10	high-ranking
**Popeye**	*Sapajus apella*	m	16	high-ranking
**Kolette**	*Sapajus apella*	f	18	low-ranking

ID Subject=Name of each individual tested, Species=Latin denomination of the species of the individual, Sex of the individual (f: female, m: male), Rank=Hierarchical within-group status determined in this study. All animals were born in captivity.

Table S3: Number of subjects per each study phase.

**Phase**	**Number of subjects *M. tonkeana***	**Number of subjects *M. fascicularis***	**Number of subjects *S. apella***	**Number of total subjects**
Food preference test	8	6	8	22
1st step Habituation Phase	8	6	8	22
2nd step Habituation Phase	6	4	7	17
Test Phase	5	3	6	14

Number of subjects that participated to the Food preference test, the Habituation Phase and finally to the Test Phase.

Table S4: Matman matrix with the order of fruit preference for the three species.

M. tonkeana								
	**raisin**	**mango**	**pineapple**	**pear**	**banana**	**orange**	**avocado**	**melon**
**raisin**	0	39	46	46	50	45	51	69
**mango**	38	0	49	49	40	39	50	53
**pineapple**	31	28	0	44	46	38	55	52
**pear**	31	28	33	0	42	39	47	60
**banana**	27	37	31	35	0	41	47	48
**orange**	32	38	39	38	36	0	50	51
**avocado**	26	27	22	30	30	27	0	39
**melon**	8	24	25	17	29	26	38	0
M. fascicularis								
	**banana**	**orange**	**pear**	**mango**	**pineapple**	**kiwi**	**apple**	**watermelon**
**banana**	0	35	39	38	44	37	50	44
**orange**	25	0	34	30	43	36	46	52
**pear**	21	26	0	33	31	32	35	54
**mango**	22	30	27	0	35	31	36	41
**pineapple**	16	17	29	25	0	38	37	40
**kiwi**	23	24	28	29	22	0	36	34
**apple**	10	14	25	24	23	24	0	36
**watermelon**	16	8	6	19	20	26	24	0
S. apella								
	**watermelon**	**mango**	**pear**	**pineapple**	**apple**	**kiwi**	**banana**	**orange**
**watermelon**	0	49	46	56	53	61	61	51
**mango**	31	0	45	39	32	40	52	51
**pear**	32	35	0	48	49	43	42	63
**pineapple**	24	41	32	0	41	42	43	54
**apple**	27	31	48	39	0	47	55	58
**kiwi**	19	40	37	38	33	0	44	56
**banana**	19	28	38	37	25	36	0	47
**orange**	29	29	17	26	22	24	33	0

Rank order of the eight fruits and the correspondent linear hierarchy index for dominance matrix in the three species. The numbers correspond to the number of times a fruit has been chosen per each couple of fruit.

Table S5: Choice-ratio of the most and least preferred fruit in long-tailed macaque and capuchin monkeys.

**ID *M. fascicularis***	**orange**	**watermelon**
Zorro	10	0
Faneliette	8	2
Carpette	8	2
Laeticiette	8	2
Nicolette	9	1
Laurencette	9	1

**ID *S. apella***	**pear**	**orange**
Willow	8	2
Popeye	10	0
Koli	10	0
Franklin	10	0
Litchi	7	3
Kolette	7	3
Kiri	7	3
Kinika	6	4

Sum of choices of the two couple of fruit which respect the minimum choice-ratio 7:3 (the total of possible choices was 10).

Table S6: Fruit used in each task/season in the three species.

**Species**	**Clumped vs. Scattered Task 1**	**Clumped vs. Scattered Task 2**	**Clumped vs. Quality Task**
M. tonkeana	pear	banana	mango (+) & orange (-)
M. fascicularis	pear	mango	orange (+) & watermelon (-)
S. apella	mango	pineapple	pear (+) & orange (-)

(+) most preferred fruit, (-) least preferred fruit

Table S7: Results of pairwise post-hoc comparisons.

Model "Successive five visited boxes CvS"

	S. apella (scattered)	*S. apella* (clumped)	*M. fascicularis* (scattered)	*M. fascicularis* (clumped)	*M. tonkeana* (scattered)	*M. tonkeana* (clumped)	Null hypothesis
S. apella (scattered)		**<0.001**					**<0.001**
S. apella (clumped)	14.676						**<0.001**
M. fascicularis (scattered)	1.210			**<0.001**			**<0.001**
M. fascicularis (clumped)			14.676				**<0.001**
M. tonkeana (scattered)	4.568		2.582			**<0.001**	**<0.001**
M. tonkeana (clumped)					14.676		**<0.001**
Null hypothesis	-7.374	9.548	-5.072	8.366	-3.373	15.282	

Model "Goal-directed strategy"

	CvS1 (scattered)	CvS1 (clumped)	CvS2 (scattered)	CvS2 (clumped)	CvQ (scattered)	CvQ (clumped)	
CvS1 (scattered)		**0.001**	**<0.001**		0,853		
CvS1 (clumped)	-3,186289			0,228		0,052	
CvS2 (scattered)	-4,9123629			**0,009**	**<0.001**		
CvS2 (clumped)		1,2050618	2,5835779			**0,002**	
CvQ (scattered)	0,1854808		4,7102354			**<0.001**	
CvQ (clumped)							

	S. apella	*M. fascicularis*	*M. tonkeana*
S. apella		**0,005**	**<0.001**
M. fascicularis	-2,806		**0,017**
M. tonkeana	-6,453	-3,142	

P-values and associated statistics (i.e. z-statistics) were obtained based on GLMMs results (see “Statistical analysis”). Grey background indicates p-values, while white background refers to the statistics. Significant p-values after correction using Benjamini and Liu's procedure are highlighted in bold. Columns and lines names indicate the situation that are compared, with parenthesis specifying within each situation, whether we compared results of visits of the clumped or scattered distribution respectively.

Table S8: Nutritional value of each of the fruit used in the tasks with the three species.

(cal/100g)

Fruit	Energy value for 100 g (cal)
Watermelon	30
Orange	47
Pineapple	50
Pear	55
Mango	60
Banana	89

Table S9: dfbetas of equivalent GLMs.

**Model « First visited box CvS »**			
	estimate	min	max
(Intercept)	-0,215	-0,259	-0,175
Context (CvS2)	0,296	0,277	0,309
Species (*M. fascicularis*)	0,272	0,225	0,308
Species (*M. tonkeana*)	0,374	0,348	0,401
Number of competitors at the starting point	0,071	0,040	0,107
Rank (Subordinate)	0,349	0,328	0,369
Day time (Morning)	-0,152	-0,170	-0,131
Session	0,103	0,092	0,117
**Model « First visited box CvQ »**			
	estimate	min	max
(Intercept)	-0,931	-0,971	-0,872
Context (CvQ)	0,027	0,010	0,045
Species (*M. fascicularis*)	1,662	1,603	1,691
Species (*M. tonkeana*)	0,457	0,424	0,485
Number of competitors at the starting point	0,121	0,045	0,156
Rank (Subordinate)	1,025	0,995	1,041

Day time (Morning)	-0,083	-0,109	-0,059
Session	0,147	0,133	0,170
**Model « Successive five visited boxes CvS »**			
	estimate	min	max
(Intercept)	-2,174	-2,199	-2,146
Context (CvS2)	-0,076	-0,084	-0,067
Rank (Subordinate)	0,157	0,145	0,171
Species (*M. fascicularis*)	0,132	0,106	0,158
Species (*M. tonkeana*)	0,863	0,843	0,880
Number of competitors at the box z	-0,264	-0,274	-0,248
Distribution of previous box (Clumped)	4,398	4,390	4,402
Day time (Morning)	-0,056	-0,070	-0,043
Session	0,161	0,153	0,168
**Model « Successive five visited boxes CvQ »**			
	estimate	min	max
(Intercept)	-2,237	-2,258	-2,205
Context (CvQ)	-0,345	-0,355	-0,335
Rank (Subordinate)	0,536	0,521	0,552
Species (*M. fascicularis*)	0,401	0,377	0,441
Species (*M. tonkeana*)	0,433	0,414	0,450
N. of competitors at the box	-0,010	-0,024	0,002
Distribution of previous box (Clumped)	4,665	4,652	4,669
Day time (Morning)	-0,110	-0,126	-0,096
Session	0,080	0,072	0,087
**Model « Goal-directed strategy »**			
	estimate	min	max
(Intercept)	-1,533	-1,554	-1,498
Species (*M. fascicularis*)	-0,980	-0,989	-0,956
Species (*M. tonkeana*)	-2,273	-2,282	-2,253
Distribution of box (Clumped)	-0,863	-0,883	-0,841
Context (CvS2)	-1,653	-1,675	-1,615
Context (CvQ)	0,137	0,115	0,150
Rank (Subordinate)	-0,255	-0,267	-0,247
Day time (Morning)	-0,075	-0,095	-0,066
Session	-0,296	-0,303	-0,289
Context (CvS2):Distribution of box (Clumped)	1,840	1,802	1,863
Context (CvQ):Distribution of box (Clumped)	-1,132	-1,155	-1,092
**Model « Change of the distribution »**	estimate	min	max
	-1,931	-1,947	-1,912
(Intercept)	-0,150	-0,160	-0,135
Species (*M. fascicularis*)	-0,368	-0,376	-0,359

Species (*M. tonkeana*)	-0,025	-0,033	-0,017
Context (CvS2)	-0,487	-0,495	-0,480
Context (CvQ)	1,435	1,418	1,461
Nature of previous box (Non-baited)	-0,631	-0,637	-0,623
Distribution of previous box (Clumped)	0,183	0,176	0,189
Rank (Subordinate)	0,068	0,058	0,074
Day time (Morning)	0,030	0,025	0,034
Session	-1,931	-1,947	-1,912

Table S10: Stability of estimates of the GLMMs.

**Model "Successive five visited boxes CvS"**	ORIG	MIN	MAX
(Intercept)	-2,207	-2,597	-1,684
Context (CvS2)	-0,156	-0,297	0,004
Rank (subordinate)	0,096	-0,293	0,430
Species (*M. fascicularis*)	0,400	-0,655	0,832
Species (*M. tonkeana*)	1,273	0,860	1,954
Number of competitors at the box z	-0,210	-0,284	-0,154
Distribution of previous box (clumped)	4,334	4,187	4,557
Day time (morning)	-0,076	-0,130	-0,004
Session z	0,152	0,082	0,202

**Model "Successive five visited boxes CvQ"**	ORIG	MIN	MAX
Intercept	-2,366	-2,907	-1,652
Context (CvQ)	-0,360	-0,499	-0,255
Rank (Subordinate)	0,523	0,014	0,945
Species (*M. fascicularis*)	0,489	-0,721	1,208
Species (*M. tonkeana*)	1,283	-0,938	1,797
N. of competitors at the box	0,024	-0,092	0,121
Distribution of previous box (Clumped)	4,638	4,420	4,847
Day time (Morning)	-0,128	-0,237	-0,002
Session	0,051	0,010	0,111

**Model « Goal-directed stategy »**	ORIG	MIN	MAX
(Intercept)	-1,362	-1,709	-1,149
Species (*M. fascicularis*)	-1,116	-1,257	-0,688
Species (*M. tonkeana*)	-2,945	-3,460	-2,440
Box distribution (clumped)	-0,983	-1,180	-0,753
Context (CvS2)	-1,598	-1,790	-1,362
Context (CvQ)	0,069	-0,096	0,272
Rank (subordinate)	-0,486	-0,677	-0,181
Day time (morning)	-0,037	-0,162	0,115
Session z	-0,361	-0,405	-0,324

Box distribution (clumped): Context (CvS2)	2,001	1,626	2,418
Box distribution (clumped): Context (CvQ)	-1,045	-1,472	-0,698

**Model « Change of the distribution »**	ORIG	MIN	MAX
(Intercept)	-2,075	-2,270	-1,599
Species (*M. fascicularis*)	-0,259	-0,416	-0,158
Species (*M. tonkeana*)	-0,463	-1,255	-0,259
Context (CvS2)	0,131	0,007	0,247
Context (CvQ)	-0,483	-0,559	-0,400
Nature of previous box (Non-baited)	1,453	1,282	1,667
Distribution of previous box (Clumped)	-0,740	-0,927	-0,438
Rank (Subordinate)	0,276	-0,083	0,475
Day time (Morning)	0,136	0,059	0,233
Session	0,077	0,046	0,104

Supplementary Figures

Figure S1a:

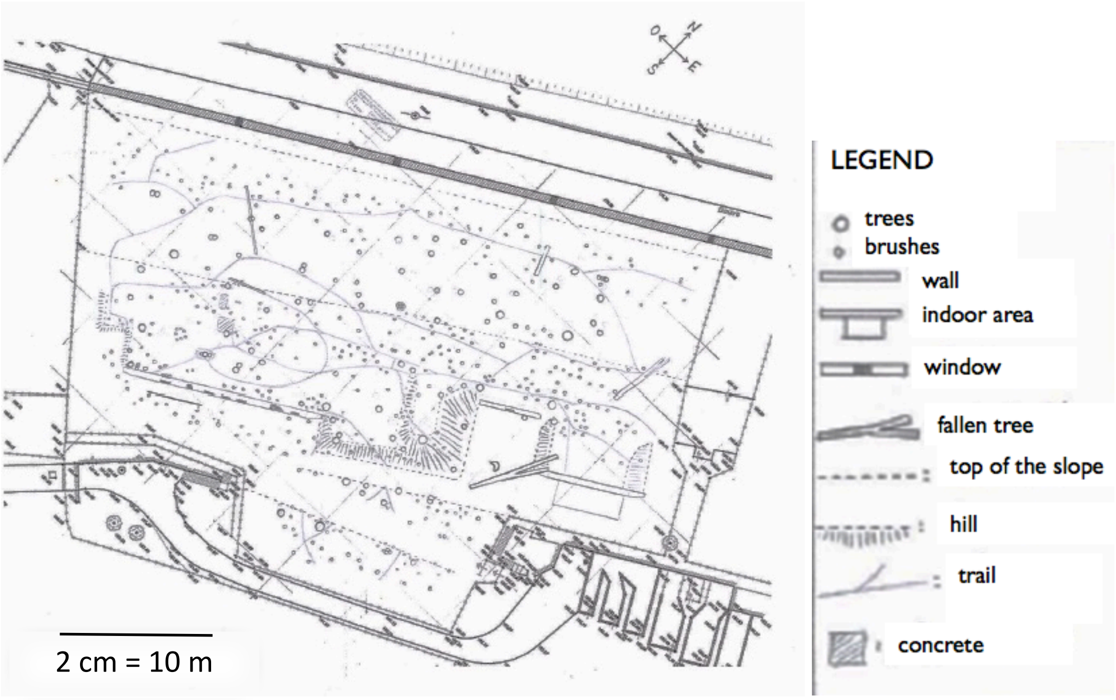

M. tonkeana outdoor area (grid of 10mx10m, 2cm=10m).

Figure S1b:

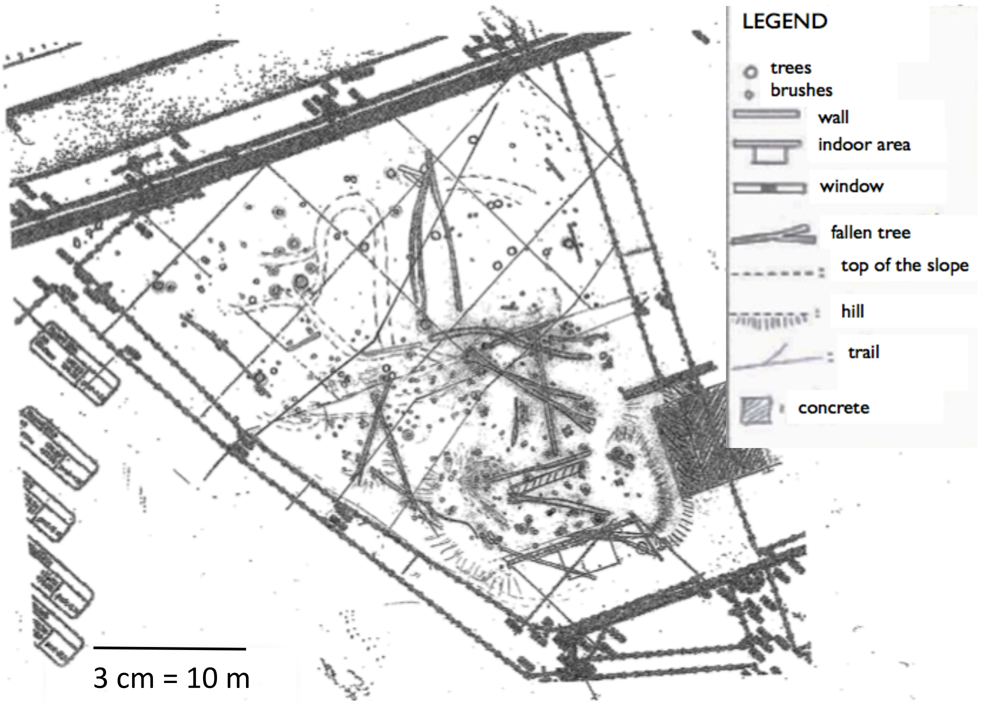

M. fascicularis outdoor area (grid of 10mx10m, 3cm=10m).

Figure S1c

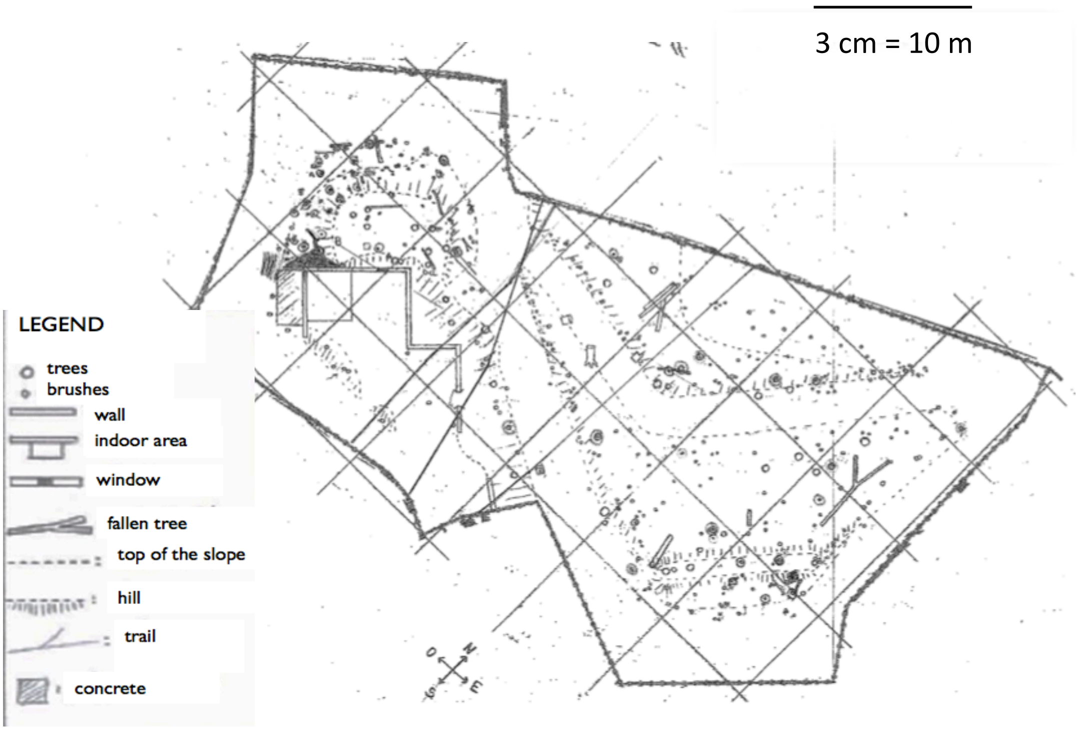

S. apella outdoor area (grid of 10mx10m, 3cm=10m).

Figure S2a:

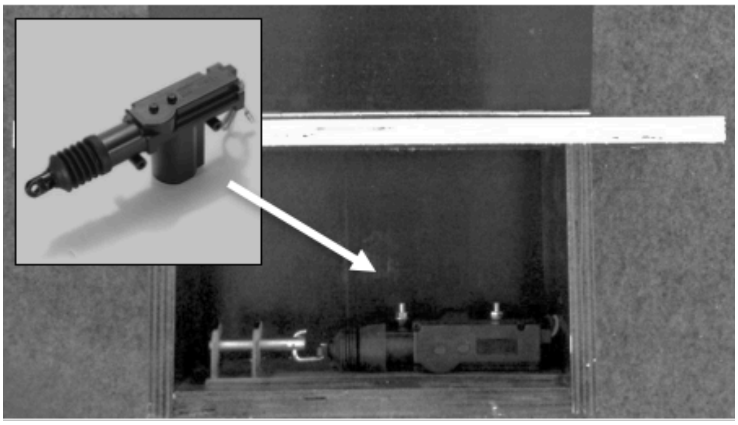

A remote-controlled box: the locker system was fixed inside the box and thus it was not visible to the monkeys from the outside.

Figure S2b:

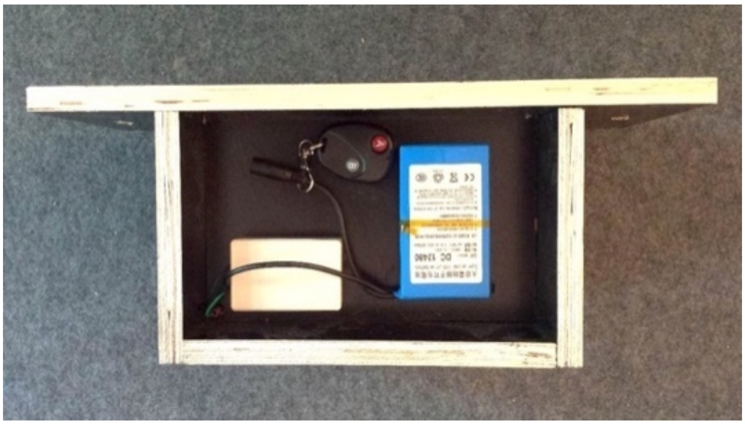

The box locker system worked through a rechargeable battery and a remote control.

Figure S3:

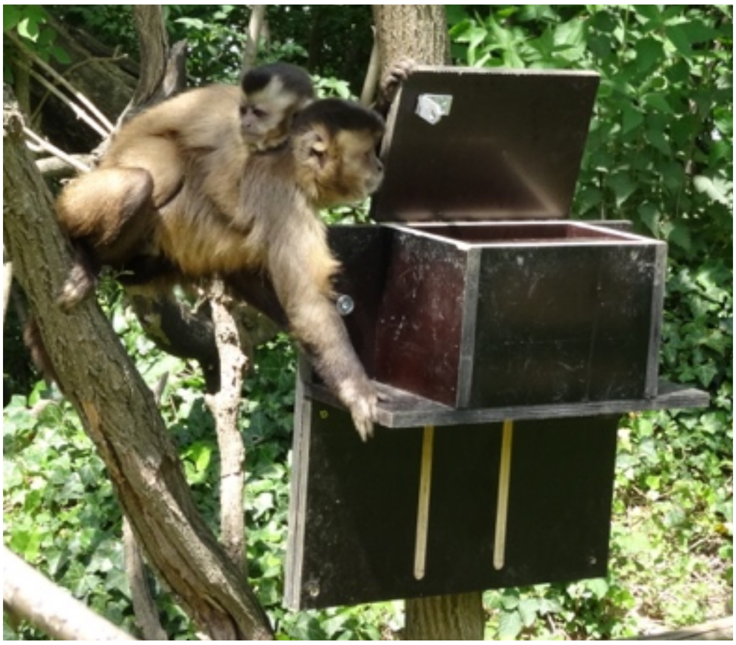

Example of a box with the correspondent *visual cue* (wooden panel engraved with an identifier combination of simple traits) fixed on a tree in *S. apella* outdoor area.

Figure S4:

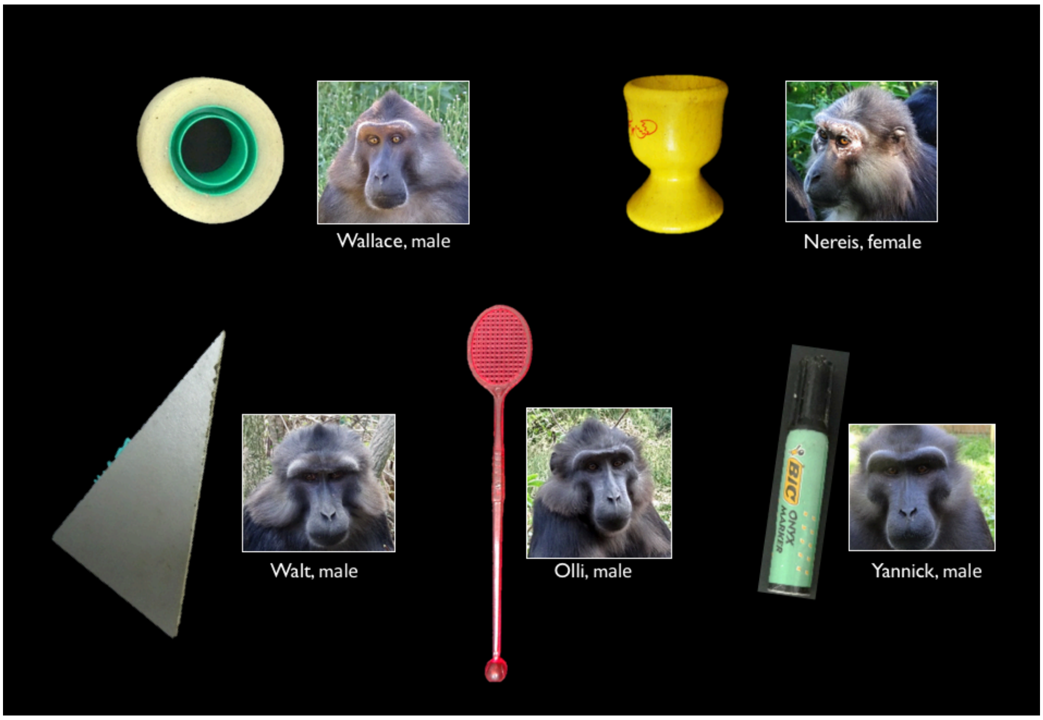

Example of *personal starting cues* used with *M. tonkeana*: a different object has been used for each individual as a signal of the beginning of the Test.

Figure S5:

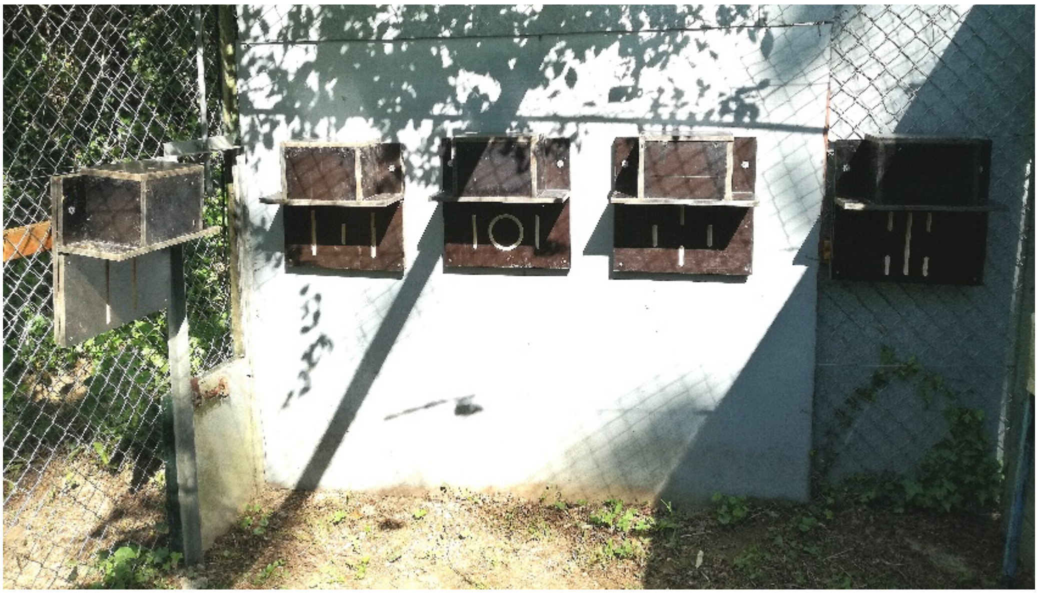

Five boxes, one for each task/season of the Test Phase, each with its corresponding visual cue fixed underneath, were placed in the experimental room of each of the study group.

Supplementary Bibliography

S1. Leca JB, Fornasieri I, Petit O. 2002 Aggression and Reconciliation in *Cebus capucinus*. Int. J. Primatol. 23(5), 979–998.

S2. Thierry B, Bynum EL, Baker S, Kinnaird MF, Matsumura S, Muroyama Y, O'Brien TG, Petit O, Watanabe K. 2000 The social repertoire of Sulawesi macaques. Primate Res. 16, 203–226.

S3. de Vries H. 1993 The rowwise correlation between two proximity matrices and the partial rowwise correlation. Psychometrika 58, 53–69.

S4. de Vries H. 1995 An improved test of linearity in dominance hierarchies containing unknown or tied relationships. Anim. Behav. 50, 1375–1389.

S5. Laska M, Hernandez Salazar LT, Rodriguez Luna E. 2000 Food preferences and nutrient composition in captive spider monkeys, Ateles geoffroyi Int J Primat 21, 671–683.

S6. Supriatna J. 2000 Primates of Indonesia. Jakarta, Indonesia.

S7. Fooden J. 1995 Systematic review of Southeast Asia long-tail macaques, *Macaca fascicularis Raffles* (1821). Fieldiana Zool. 64, 1–44.

S8. Cunha AA, Vieira MV, Grelle CEV. 2006 Preliminary observations on habitat, support use and diet in two non-native primates in an urban Atlantic forest fragment: The capuchin monkey (*Cebus* sp.) and the common marmoset (*Callithrix jacchus*) in the Tijuca forest, Rio de Janeiro. Urban Ecosys. 9(4), 351–359. (https://doi.org/10.1007/s11252-006-0005-4)
